# Clinical utility of therapeutic drug monitoring of sertraline: a transdiagnostic systematic scoping review

**DOI:** 10.3389/fphar.2026.1812662

**Published:** 2026-06-29

**Authors:** Covadonga Canga-Espina, José Pablo Bullard, Begoña Tapia-Alzuguren, Adam Gędek, Enrique Aubá, Felipe Ortuño, Azucena Aldaz-Pastor, Patricio Molero

**Affiliations:** 1 Department of Psychiatry and Clinical Psychology, Clínica Universidad de Navarra, Pamplona, Spain; 2 Pharmacy Services, Clínica Universidad de Navarra, Pamplona, Navarra, Spain; 3 Third Department of Psychiatry, Institute of Psychiatry and Neurology, Warsaw, Poland; 4 Navarra Institute for Health Research (IdiSNA), Pamplona, Spain

**Keywords:** depressive disorder, obssessive-compulsive disorder, personalized medicine, sertraline, therapeutic drug monitoring (TDM)

## Abstract

**Introduction:**

Therapeutic drug monitoring (TDM) is the quantification and interpretation of drug concentrations in blood to personalize pharmacotherapy. Dose adjustment of sertraline is currently based on clinical judgment. Preliminary evidence shows relationships between sertraline blood concentration and clinical outcomes. This review aims to evaluate the clinical utility of TDM of sertraline.

**Methods:**

A comprehensive, systematic search of studies in several databases was conducted up to 2025/06/26 on the potential relationship between TDM of sertraline and measures of efficacy, safety and tolerability. The search identified 5 clinical trials and 5 observational studies (N = 1106).

**Results:**

The included studies are highly heterogeneous. Most of the results are observational, mainly focused on depression and obsessive-compulsive disorder, covering the whole dosage range, with populations representing the whole life-span, observational periods from 4 weeks to 1 year. Efficacy assessments utilized diverse scales and heterogeneous measures of safety/tolerability. Most of the studies do not detect concentration-effect relationships. Preliminary, non-linear concentration-effect relationships are reported for efficacy and tolerability.

**Conclusion:**

There is no sufficient evidence for the clinical utility of a generalized use of TDM for sertraline. Preliminary evidence suggests associations between blood concentrations of sertraline and clinical outcomes, potentially supporting the utility of its TDM as guide for clinical judgement in the treatment of depression and obsessive-compulsive disorder, particularly in populations with specific risks. Further studies using symptom-specific clinical scales are needed to assess the potential clinical utility of sertraline TDM in personalized medicine.

**Systematic Review Registration:**

https://osf.io/84trm/overview.

## Highlights

- The available evidence is highly heterogeneous.
- Preliminary evidence suggests associations between blood concentrations of sertraline and clinical outcomes, potentially supporting the utility of its TDM in populations with specific risks.

## 1 Introduction

Therapeutic drug monitoring (TDM) is the quantification and interpretation of drug concentrations in blood (serum or plasma) to optimize and personalize pharmacotherapy (Hiemke et al., 2018). TDM is a tool for precision medicine due to its contribution to the prescription of the right dose for each individual patient (Jang, 2016) throughout the different pathophysiological stages of the illness’s evolution, which may imply clinically significant pharmacokinetic variability.

In psychiatry, TDM is a standard of care or recommended under certain clinical situations for the optimization of specialized-level treatments with mood stabilizers, antipsychotics and tricyclic antidepressants with established therapeutic and/or safety ranges, such as lithium, valproate, carbamazepine, clozapine, amisulpride, amitriptyline and nortriptyline, to mention some examples (Hiemke et al., 2018; National Institute for Health and Care Excellence, 2014; Schoretsanitis et al., 2018).

In the case of selective serotonin reuptake inhibitors (SSRIs), which are first-line pharmacological treatment for a variety of medical conditions and disorders, TDM is considered generally less useful in routine clinical practice (Taylor et al., 2025). However, growing evidence derived from the differential pharmacological properties of certain SSRIs may be worth the consideration of TDM. One example is citalopram, for which TDM was changed to the highest level of recommendation by the TDM expert group of the Arbeitsgemeinschaft für Neuropsychopharmakologie und Pharmakopsychiatrie (AGNP) guidelines (De Leon, 2018; Schoretsanitis et al., 2018), a decision supported and further informed by recent evicence (Xu et al., 2023).

Another SSRI for which TDM may be clinically useful is sertraline, given the preliminary evidence showing a possible relationship between its concentration in blood and efficacy measures for prevalent conditions such as depressive disorder (Mauri et al., 2003), the need for special attention to unexpected side effects and safety issues in adolescents and elderly patients (Shu et al., 2025) and the fact that its metabolite to parent compound ratio (N-desmethylsertraline to sertraline) may reflect treatment compliance beyond a mere quantification of sertraline in blood (Reis et al., 2004b). However, the degree and extent of the clinical utility of sertraline TDM are not clear neither in the field of efficacy, considering discrepant results (for instance, detection of concentration-efficacy relationships in some but not all studies focused on different diagnoses, such as depression and obsessive-compulsive disorder (OCD) (Mauri et al., 2002; Tini et al., 2022), or regarding tolerability, which is generally good (Cipriani et al., 2010) in comparison with other SSRIs. Sertraline is a common pharmacological treatment for several psychiatric disorders such as Major Depressive Disorder (MDD), Obsessive-Compulsive Disorder and some anxiety disorders. Dose adjustment is commonly made by clinical judgment not guided by objective analytical measures. The determination of blood levels by means of Therapeutic Drug Monitoring (TDM) may be helpful in this regard.

This review aims to evaluate the clinical utility of sertraline TDM transdiagnostically, based on primary research evidence regarding whether there are optimal ranges of blood sertraline concentrations associated with therapeutic response (efficacy), safety and tolerability in any indication. Given the amplitude, complexity and heterogeneity of the topic, a scoping review following the PRISMA-ScR guidelines (Peters et al., 2020; Tricco et al., 2018) is the most appropriate methodology as a first evidence synthesis approach to map the available evidence and identify key findings, knowledge gaps and unmet needs for future research plans (Munn et al., 2018). Our objective is to find clinical research studies on this matter not only in sertraline’s most common indication (depressive disorder), but also in other indications.

## 2 Methods

This scoping review was conducted and reported in accordance with the PRISMA Extension for Scoping Reviews (PRISMA-ScR) (Munn et al., 2018; Tricco et al., 2018). A protocol for this study was drafted and pre-registered at the Open Science Framework (OSF) (https://osf.io/84trm) (DOI: https://doi.org/10.17605/OSF.IO/84TRM) (Canga-Espina et al., 2025).

### 2.1 Search strategy and selection criteria

The search strategy was based on the PRISMA Extension for Scoping Reviews (PRISMA-ScR) quality criteria (Tricco et al., 2018) and the Joanna Briggs Institute (JBI) (Peters et al., 2020). An optimal balance between pragmatic considerations and scientific rigor was sought by focusing the systematic, comprehensive literature searches in PubMed, Web of Science and Scopus up to 26 June 2025, without language restrictions. In addition, non-systematic searches were conducted in the pre-print servers MedRxiv and PsyArXiv, as well as Scopus and Google Scholar, to identify relevant published articles and grey literature. Hand-searching of reference lists was also conducted through ascendancy and descendancy approaches. All the search strings are provided in the supplementary material (Supplementary Table S1).

Eligible studies needed to fulfill all the following inclusion criteria, according to the forementioned PCC framework (Peters et al., 2020; Tricco et al., 2018): (P) *Population*: patients under treatment with sertraline regardless of their sex, age, main diagnosis nor daily dosage; (C) *Concept*: patients receiving sertraline TDM with documented measures of efficacy, safety and/or tolerability; (C) *Context*: patients in either hospitalized or ambulatory regimen. Exclusion criteria included: studies not based on primary data (i.e., reviews, meta-analyses, expert opinions or consensus) or those that did not evaluate efficacy, safety and/or tolerability; or that did not investigate sertraline.

The focus of this scoping review was restricted exclusively to studies with specific, explicit and direct assessment of the relationship between TDM of sertraline and its therapeutic response (efficacy), safety and tolerability in any of its indications. Therefore, other aspects of TDM indirectly related to efficacy and safety such as compliance and analytical or pharmacogenetic variables (not assessed via a direct safety or tolerability exploration) were not considered.

#### 2.2 Screening, data extraction and synthesis

A first screening was based on titles, abstracts and keywords; a second one on full texts. The whole screening process and deduplications were conducted by two independent authors sequentially, using Covidence software. Inclusion was determined by consensus. Retracted articles were not included, while conference abstracts were reviewed to capture ongoing or unpublished research. In case multiple articles reported on data derived from the same cohort in different time points, we included all of the articles to capture the possible influence of the length of treatment period or stages of treatment. In case of doubt or absence of agreement regarding whether to include an article, a discussion with a third, senior author was held to reach a final consensus. Screening agreement was assessed by means of inter-rater reliability (Cohen’s kappa coefficient).

Entities extracted were: type of study, year of publication, country, N, mean age, % women, sertraline dosage, time of follow-up, domains studied (efficacy, safety, tolerability), measures of efficacy and/or safety and tolerability (scales, checklists used), diagnosis, description of the main results by domain and conclusions.

The synthesis of the extracted data was made by means of basic descriptive analysis and mapped tables (Tricco et al., 2018; Urrútia and Bonfill, 2010).

In accordance with recommendations for Scoping Reviews (Peters et al., 2020; Tricco et al., 2018; Munn et al., 2018), formal assessment of the methodological quality (sensitivity analyses, robustness checks, publication bias and risk of bias) was not carried out.

## 3 Results

### 3.1 Study selection

**FIGURE 1:**
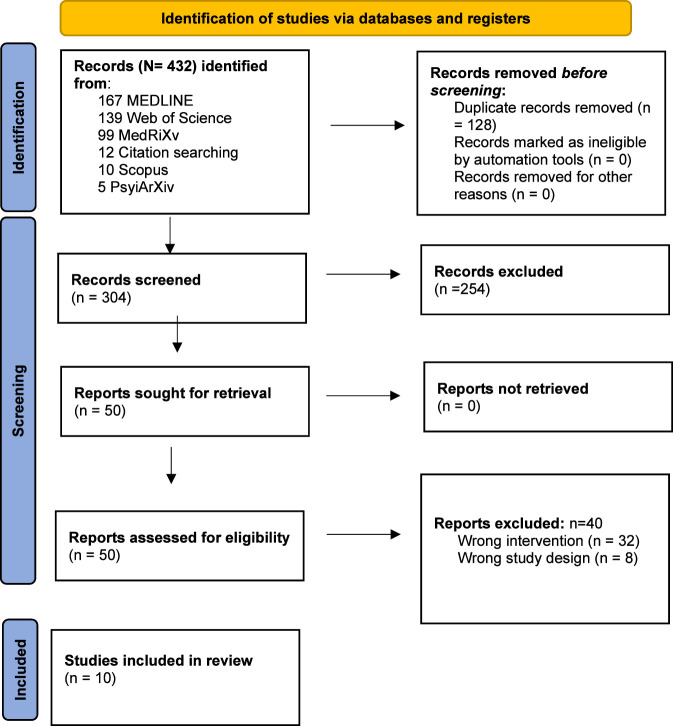
Flowchart on identification, screening and inclusion of eligible publications discharged from Covidence tool (downloaded from: https://www.prisma-statement.org/prisma-2020-flow-diagram)*.*

The initial search yielded 432 records (k), of which 12 articles were manually collected and screened. Out of these, 304 were unique records. A total of 254 records were excluded after reading title and/or abstract, leaving 50 articles for eligibility consideration. Of these, 40 articles did not meet the inclusion and exclusion criteria, leaving 10 studies that were retained and included in our data synthesis. Inter-rater reliability/agreement (Cohen’s Kappa) was moderate, and discrepancies were resolved by consensus meetings (shown in the Supplementary Table S2). Study selection is presented in Figure 1. One conference abstract was identified (Klampfl et al., 2011) that was closely aligned with one included study (Taurines et al., 2013), but provided insufficient additional information to be included as an independent study. The included studies are synthesized and presented in Table 2.

### 3.2 Study characteristics

The clinical utility of sertraline TDM has been studied in five clinical trials (Alderman et al., 1998; 2006; Greist et al., 1995a; Greist et al., 1995b; Reis et al., 2004b) (two randomized, of which one was placebo controlled, and two open label) and five observational studies (four prospective naturalistic (Lundmark et al., 2000b; Mauri et al., 2002; 2003; Tini et al., 2022) and one cross-sectional (Taurines et al., 2013)). The demographic characteristics of the included studies are shown in Table 1. The pooled data spanned a total of 1106 subjects with observational periods spanning from 4 weeks to 1 year, age range from 6 to over 80 years old, covering a variety of diagnoses of which the most represented ones are OCD (80.2%) amongst clinical trials and depression (single and recurrent episode) amongst observational studies (58.7%).

**TABLE 1:** Demographic characteristics of the included studies of TDM of sertraline.

Type of study	Age (mean, (years)	N	% females	Diagnosis (N, %)
Clinical trial^Φ^ (Alderman et al., 1998 ; 2006; Greist et al., 1995; Greist et al., 1995b; Reis et al., 2004b)	26.33*	857	53.26%	• OCD (N = 687; 80.2%) • MDD (N = 168; 19.6%) • OCD + MDD (N = 2; 0.2%)
Observational^Φ^ (Lundmark et al., 2000b; Mauri et al., 2002 ; 2003; Taurines et al., 2013; Tini et al., 2022)	41.56	249	60.24%	• Depressive Episode (N = 102; 41%) • OCD (N = 56; 22.5%) • Recurrent Major Depressive Disorder (N = 44; 17.7%) • Other anxiety disorders (N = 19; 7.6%) • ED (N = 15; 6%) • PTSD (N = 3; 1.2%) • ASD (N = 2: 0.8%) • Schizophrenia (N = 1; 0.4%) • Cyclothymic disorder (N = 1; 0.4%) • Phobic anxiety disorders (N = 1; 0.4%) • Impulse disorders (N = 1; 0.4%) • Tic Disorder (N = 1; 0.4%) • Mixed disorders of conduct and emotions (N = 1; 0.4%) • Emotional disorders with onset specific to childhood (N = 1; 0.4%) • Disorders of social functioning with onset specific to childhood and adolescence (N = 1; 0.4%)

Abbreviations: ASD: autistic spectrum disorder; DSM-III-R: diagnostic and statistical manual of mental disorders; third edition revised; DSM-IV: diagnostic and statistical manual of mental disorders; fourth edition, ED: eating disorder; ICD-10: International Classification of Diseases 10th Revision; MDD: major depressive disorder; OCD: Obsessive-Compulsive Disorder; PTSD: Post-traumatic Stress Disorder.

^*^ ((Reis et al., 2004b) used the median, so it is excluded as it is a central value of a distribution while the mean is an individual data average) (Higgins et al., 2024).

^Φ^ Diagnosed by DSM-III-R.

^֏^Diagnosed by ICD-10, or DSM-IV.

Regarding the demographic characteristics of the populations studied, the whole lifespan is represented: four studies were focused on infancy and adolescence (Alderman et al., 1998; 2006; Taurines et al., 2013; Tini et al., 2022), five on adulthood (Greist et al., 1995a; Greist et al., 1995b; Mauri et al., 2002; 2003; Reis et al., 2004b) and one in advanced age (Lundmark et al., 2000a). Females were predominant in seven samples, focused in MDD and OCD (Alderman et al., 1998; 2006; Taurines et al., 2013), depression ((Lundmark et al., 2000b; Mauri et al., 2002; Reis et al., 2004a) and a depression subsample (Tini et al., 2022); whereas males were predominant in three samples, focused on OCD (Greist et al., 1995a; Greist et al., 1995b), depression (Mauri et al., 2003) and an OCD subsample (Tini et al., 2022).

The doses studied covered the full range accepted for sertraline (25–200 mg/day), and one study extended the range up to 250 mg/day in OCD (Tini et al., 2022). TDM was carried out in plasma (6 studies) (Alderman et al., 1998; 2006; Greist et al., 1995a; Greist et al., 1995b; Mauri et al., 2002; 2003) or serum (4 studies) (Lundmark et al., 2000a; Reis et al., 2004a; Taurines et al., 2013; Tini et al., 2022) samples, using both single (3 studies) and repeated measures (7 studies) designs.

### 3.3 Clinical outcomes–efficacy

Efficacy was assessed in the included studies, through global, non-specific measures (such as the Clinical Global Impressions scale improvement subscale (CGI-I) (Overall and Gorham, 1962) and Severity subscale (CGI-S) (Guy, 1976), the Global Effectiveness Evaluation (GEE)) and specific clinician-administered clinical scales (the Montgomery-Asberg Depression Rating Scale (MADRS) (Montgomery and Åsberg, 1979), the Hamilton depression Rating Scale (HDRS, (Hamilton, 1960), the Hamilton anxiety Rating Scale (HARS) (Hamilton, 1959), the Y-BOCS scale (Goodman, 1989), the Maudsley Obsessive-Compulsive Inventory (MOC) (Hodgson and Rachman, 1977) and the Brief Psychiatric Rating Scale (BPRS) (Overall and Gorham, 1962).

Two observational studies reported concentration-therapeutic effect relationships: a statistically significant curvilinear relationship between the range of 25–50 ng/mL and improvement of depressive symptoms measured by the HDRS in a study of 23 adults with recurrent MDD (mean age 52.5 years) followed up for 12 months (Mauri et al., 2003), and an estimation of effective concentration reference levels of 66–76 ng/mL (25th–75th interquartile) for clinical improvement measured by the CGI-TE in an OCD subsample of 21 children and adolescents (mean age: 13.9 years) followed up for at least 6 months (Tini et al., 2022).

The remaining eight studies, encompassing 810 adults, with observational periods from 3 to 12 months, and 194 children and adolescents, with observational periods from 5 weeks to 12 months, did not detect significant concentration-therapeutic effect relationships. Of these studies focused on depression, only one (Reis et al., 2004b) also used a specific scale for depressive symptoms (MADRS) in the long term (6 months) for the evaluation of therapeutic effects; five studies used the non-specific CGI (Alderman et al., 1998; 2006; Taurines et al., 2013; Tini et al., 2022) and Global Effectiveness Evaluation (GEE) (Lundmark et al., 2000a) scales, and one other study also using the HDRS had an observation period of 4 weeks (Mauri et al., 2002). In the case of OCD, none of the studies using specific scales (Alderman et al., 1998; 2006; Greist et al., 1995a; Greist et al., 1995b) detected any concentration-therapeutic effect relationships (Table 2).

**TABLE 2:** Main characteristics of the included studies of TDM of sertraline (in chronological and alphabetical order).

First author, year	Country	Study type	Follow up	N, diagnosis	Mean age (years)	% Female	Dosage (mg/day)	Methods			Results	
								TDM (single or repeated)	Efficacy	Safety/Tolerability	Efficacy	Safety/Tolerability
Greist et al. (1995b)	United Stated	Clinical trial, randomized, double-blind, placebo-controlled parallel of three fixed doses	12 weeks	324: OCD (DSM-III-R) (baseline NIMH-OC≥7 and HDRS≤17)	39.6	40 (mean sample)	50, 100 and 200	Plasma repeater measures	Y-BOCS, NIMH-OC, CGI-S, CGI-I, HDRS	All volunteered or observed adverse events were recorded (WHO dictionary preferred terminology)	No concentration-effect relationship detected	No concentration-side effect relationship reported
Greist et al. (1995)	United States	Clinical trial, randomized, double-blind, placebo-controlled parallel of three fixed doses	1 year: 12 weeks and additional 40 weeks extension	336 total: 240 (12 weeks); 96 (40 weeks extension), OCD (DSM-III-R)	39.6	41.2 (total sample)	50, 100 and 200	Plasma, repeated measures	Y-BOCS, NIMH-OC, CGI-S, CGI-I, MOC	All volunteered or observed adverse events were recorded, physical exploration, LT, weight, ECG	No concentration-effect relationship detected	No concentration-side effect relationship reported
Alderman et al. (1998)	United States	Clinical trial, open label, non-placebo-controlled multicenter	5 weeks	61 total: MDD (44), OCD (16) MDD + OCD (1) (DSM-III-R)	12.8	61	50–200	Plasma, repeated measures	MDD: CGI-I, CGI S OCD: CY-BOCS NIMH- OCD CGI-I, CGI-S	All volunteered or observed adverse events were recorded, physical exploration, LT, weight, ECG	No concentration-effect relationship detected	No concentration-side effect relationship detected
Lundmark et al. (2000a)	Sweden	Observational, prospective, naturalistic, cohort study Single-center	6–9 months	37: Depressive Episode (ICD-10)	76	78	25–100	Serum, single measure	GEE	“Direct questioning”	No concentration-effect relationship detected	No concentration-side effect relationship detected
Mauri et al. (2002)	Italy	Observational, prospective, naturalistic, single-center, cohort study	4 weeks	21: Recurrent Major Depressive Disorder (DSM-IV)	50.23	66,7	25–150	Plasma, single measure	BPRS HARS HDRS	Checklist	No concentration-effect relationship detected	No concentration-effect relationship detected
Mauri et al. (2003)	Italy	Observational, Prospective, naturalistic, single-center, cohort study	12 months	23: Recurrent Major Depressive Disorder (DSM-IV)	52.56	39,1	25–150 (x̄ = 62,79 ± 34.02)	Plasma, repeated measures	BPRS HARS HDRS	Checklist	Concentration-effect relationship (curvilinear, 25–50 ng/mL)	No concentration-effect relationship detected
Reis et al. (2004b)	Sweden	Clinical trial, randomized (sertraline vs. paroxetine), non-placebo controlled open-label, multicentric	6 months	92: MDD (DSM-III-R) (baseline MADRS≥21)	42 (median)	66	50–150	Serum, repeated measures	MADRS CGI	Not evaluated	No concentration-effect relationship detected	Not evaluated
Alderman et al. (2006)	United States	Clinical trial, open label, non-placebo-controlled multicenter (extension study of Alderman et al. 98)	6 months	43: MDD (32); OCD (10) MDD + OCD (1) (DSM-III-R)	13.3	58.1	50–200 (x̄ = 157 ± 49)	Plasma, repeated measures	MDD: CGI-I, CGI S OCD: CY-BOCS NIMH- for OCD CGI-I, CGI-S	All volunteered or observed adverse events were recorded, lab tests, weight, physical exam, ECG	No concentration-effect relationship detected	No concentration-side effect relationship reported
Taurines et al. (2013)	Germany	Observational, cross-sectional, multicenter, naturalistic	-	90 total OCD (35), Depressive Episode (30), other diagnosis non-specified (17), Eating Disorder (8) (ICD-10)	14.8	53.9	25–200 (x̄ = 83.5)	Serum, single measure	CGI-I	UKU-SERS	No concentration-effect relationship detected	Whole sample: No concentration-effect relationship detected; Subsample with Depressive Episode: higher concentrations (mean 44.8 ng/mL) associated with side effects
Tini et al. (2022)	Germany, Austria Switzerland	Observational, prospective, naturalistic, multicentric	At least 6 months	78 total Depressive Episode (35) OCD (21), ED (7), PTSD (3), ASD (2), other anxiety disorders (2), schizophrenia (1), Cyclothymic disorder (1), Phobic anxiety disorders (1), impulse disorders (1), Tic Disorder (1), Mixed disorders of conduct and emotions (1), emotional disorders with onset specific to childhood (1), Disorders of social functioning with onset specific to childhood and adolescence (1) (ICD-10)	Globally: 14.22 14.5 (Depressive Episode) 13.9 (OCD)	62.8 (all sample), 75% (Depressive Episode) 47,6% (OCD)	25–200 (Depressive Episode) 50–250 (OCD)	Serum, repeated measures	CGI-TE	CGI-SE PAERS	Whole sample and Depressive Episode subsample: No concentration-effect relationship detected OCD subsample: concentration-effect relationship (66–76 ng/mL)	No concentration-effect relationship detected

Abbreviations: ASD: autistic spectrum disorder, BPRS: brief psychiatric rating scale; CGI-I: Clinical Global Impressions Scale- - Improvement subscale; CGI-TE: Clinical Global Impression–Therapeutic Effects; CGI-S: Clinical Global Impressions Scale-Severity subscale; CGI-SE: Clinical Global Impressions Scale - Side Effects subscale; CY-BOCS: Children Yale-Brown Obsessive-Compulsive Scale; DSM-III-R: diagnostic and statistical manual of mental disorders; third edition revised; DSM-IV: diagnostic and statistical manual of mental disorders; fourth edition, ECG: electrocardiogram; ED: eating disorder; GEE: global effectiveness evaluation; HARS: hamilton rating scale anxiety subscale; HDRS: hamilton rating scale depression subscale; LT: laboratory tests; ICD-10: International Classification of Diseases 10th Revision; MADRS: Montgomery-Asberg Depression Rating Scale; MDD: major depressive disorder; MOC: Maudlsey Obsessive-Compulsive Inventory, NIMH-OCD: National Institute of Mental Health Scale for Obsesive-Compulsive Disorder; OCD: Obsessive-Compulsive Disorder; PAERS: pediatric adverse event rating scale; PTSD: Post-traumatic Stress Disorder; UKU-SERS: Udvalg for Kliniske undersøgelser side effect rating scale; Y-BOCS: Yale-Brown Obsessive-Compulsive Scale.

### 3.4 Clinical outcomes–safety and tolerability

Safety and tolerability were assessed in 9 studies, through recording of volunteered or observed adverse effects, checklists, specific scales (2 studies: the Udvalg for Kliniske undersøgelser side effect rating scale (UKU-SERS) (Lingjærde et al., 1987) and the Pediatric Adverse Event Rating Scale (PAERS) (Leung et al., 2022; Wehmeier et al., 2008) and in 4 studies also through lab tests, physical exam and ECG (Table 2).

A cross-sectional study found higher concentrations (44.8 ng/mL *versus* 22.3 ng/mL) associated with side effects measured by the UKU-SERS in a subsample of 30 children and adolescents (mean age: 14.8 years) with MDD (Taurines et al., 2013). The remaining 8 studies did not detect concentration-side effect relationships. None of these studies used the UKU-SERS for the evaluation of side effects.

None of the 5 clinical trials detected concentration-effect relationships (only 3 of them included the pharmacokinetics of sertraline as a primary outcome (Alderman et al., 1998; 2006; Reis et al., 2004b). Briefly, their results beyond the question of interest of this review were the following ones: one study supported the efficacy and safety of three doses of sertraline for OCD (50, 100 and 200 mg/day) in adults over 12 weeks (Greist et al., 1995b), with extension of this evidence throughout 1 year of treatment in a continuation study (Greist et al., 1995a); one clinical trial focused on the pharmacokinetics of sertraline in pediatric patients proved the safety and efficacy of the adult titration schedule over 5 weeks for children and adolescents with depression and OCD (Alderman et al., 1998), with extension of this evidence throughout 6 months of treatment in a continuation study (Alderman et al., 2006); and the pharmacokinetic part of a large phase IV clinical trial comparing sertraline and paroxetine found significant interindividual variability of the serum levels of sertraline with intraindividual stability over 6 months (Reis et al., 2004b).

## 4 Discussion

Sertraline is among the most commonly prescribed psychiatric medications and is used across multiple psychiatric indications, with a wide therapeutic dosing range (from 25 to 200 mg/day and higher in off-label settings). The dose range is mainly non-indication-specific, other than lower starting dosage for anxiety disorders and posttraumatic stress disorder, and lower maximum dosage for premenstrual dysphoric disorder (FDA). However, beyond clinical response, there are currently limited tools available to support dose individualization and treatment optimization in routine clinical practice. To our knowledge, this is the first systematic scoping review on the clinical utility of sertraline TDM, focused on efficacy, safety and tolerability measures adopting a transdiagnostic approach throughout the life span.

This review did not find a consistent association between sertraline blood concentration and clinical outcomes of efficacy and safety and/or tolerability. The studies directly assessing this relationship are highly heterogeneous regarding the diagnoses included, methods of TDM and clinical assessment, demographic characteristics of the populations included and study designs. The absence of a concentration-therapeutic effect relationship for sertraline may be due to pharmacodynamic reasons, given that relatively low concentrations of sertraline, commonly corresponding to medium or even low doses, already achieve a significant proportion (around 80%) of serotonin transporter (SERT) occupancy in the brain (Hart et al., 2024). Of note, these concentrations are in the order of (or even below) the low threshold of the current recommended therapeutic reference range (10 ng/mL, (Hiemke et al., 2018)). The absence of concentration-side effect relationships may be due to the generally good tolerability profile of sertraline at the recommended doses (Cipriani et al., 2010). The apparent scarcity of studies on the association between sertraline blood levels and clinical outcomes may be explained by the notion of a predicted response in typical populations characterized by a wide effective dose range with a flat dose-SERT occupancy curve, together with an also wide separation between the toxicity alert level (300 ng/mL) and the upper threshold of blood levels commonly reached in the recommended doses (150 ng/mL) (Hiemke et al., 2018), in contrast with other drugs such as lithium or clozapine.

Despite of the methodological heterogeneity and discrepant results, this review reveals trends of possible age and diagnosis-specific relationships between blood concentrations of sertraline and both therapeutic and side effects in MDD and OCD. These are observed when assessed by clinician-administered scales of sufficiently broad spectrum coverage of specific clinical manifestations and side effects, such as the HDRS (Mauri et al., 2003) or UKU-SERS (Taurines et al., 2013) respectively, and during observational periods covering a significant portion of the treatment period depending on the diagnosis (6–12 months). However, absence of multiple testing correction is a limitation of these results. These conditions may be relevant in the investigation of the potential clinical utility of sertraline TDM.

### 4.1 Method of TDM

The blood concentration of sertraline was assessed both in plasma and in serum. This difference may be theoretically relevant, as sertraline has high protein binding (circa 98%) (Apseloff et al., 1997). Plasma requires anticoagulants, which can affect the binding of the drug to proteins; while serum is obtained after the liberation of proteins to reach coagulation, which can also affect the binding of the drug to the proteins but in a different way. These factors can affect the analysis of drug levels in blood; however, to date, no such changes have been found in the use of antidepressants and specifically of sertraline (Uges, 1988; Yu et al., 2011). In this scoping review, we did not observe any clear patterns indicating that one of the methods was more useful than the other. This is in agreement with preliminary evidence supporting the notion that both techniques yield equivalent results (Hiemke et al., 2018).

### 4.2 Clinical assessments in the study of TDM

The results of this review highlight the importance of the clinical scales selected to assess the clinical utility of sertraline TDM for each diagnosis. In the case of depression, none of the studies that assessed efficacy with global, non-specific scales such as the CGI and GEE detected concentration-effect relationships. The only concentration-effect relationship was found using the HDRS, a validated clinician-administered scale for monitoring the intensity of depressive symptoms. The fact that a study using the MADRS (Reis et al., 2004b), also validated to detect the treatment response of depressive symptoms, did not find a concentration-effect relationship might be due to differences between the HDRS (more influenced by anxiety and physical symptoms) and the MADRS (more focused in core depressive symptoms) in their design, sensitivity and psychometric properties. Interestingly, the only study that systematically measured the presence of side effects through the UKU-SERS (Taurines et al., 2013) was the one finding a possible concentration-side effects association in the depressive episode subsample. The UKU-SERS can be considered the gold standard for a systematic, comprehensive assessment of side effects of psychotropic medications in clinical research, covering a wide range of clinician-rated psychic and physical side effects (Lingjærde et al., 1987). As mentioned above, an important caveat in this regard is that multiple testing correction should be taken into account in the analysis of different symptomatic profiles with specific scales.

### 4.3 The role of TDM in different stages of treatment

TDM may have different clinical utility in different stages of treatment. The only concentration-therapeutic effect findings have been observed in long-term treatment stages: a statistically significant association of the range 25–50 ng/mL with improvement in adults with recurrent MDD (12 months follow-up) (Mauri et al., 2003), and tentative effective concentration reference levels of 66–76 ng/mL (25th–75th interquartile) in good responders children and adolescents with OCD with a minimum of 6 months follow-up (Tini et al., 2022). This is of interest given the current recommendation of using the interquartile range of responders in naturalistic studies (Hart et al., 2025; Hiemke, 2019; Preskorn, 2014). There is only one observation in a shorter-term treatment stage (30 days of follow-up), also in adults with MDD (Mauri et al., 2002), according to which in spite of the absence of correlation between sertraline plasma levels and clinical improvement or side effects, a statistically significant curvilinear relationship was identified between sertraline plasma levels and specific affective cluster scores measured by the BPRS (blunted affect and emotional withdrawal), with lower scores (milder symptoms) in the range between 40 and 70 ng/mL. This contrast between the shorter and longer terms leads to the hypothesis of whether specific intermediate ranges of blood concentrations of sertraline are associated with efficacy as a function of specific symptom profiles, as opposed to total scores of clinical scales, especially in the first stages of the treatment of MDD. These findings support the potential clinical utility of sertraline TDM in the treatment optimization of different stages of MDD.

### 4.4 Clinical value of TDM to assess intraindividual variability, compliance and other aspects

Given that intraindividual variability of sertraline blood concentration and the desmethylsertraline/sertraline ratio are low (Lundmark et al., 2000a; Reis et al., 2004b), additional potential clinical utilities of sertraline TDM include: i) prediction of blood concentrations after dose adjustment and assessment of drug-drug interactions; ii) determination of doses and titration regimens in different age ranges or population with special risks (children, adolescents and elderly subjects, comorbidities and polymedication); iii) finding the minimum effective dose for each individual patient, especially in the long term, to improve tolerability and drug costs reduction; and iv) the utility and feasibility of repeated measures in the long term of TDM in MDD to detect intraindividual variability of both concentration and desmethylsertraline/sertraline ratio, a consequence not only of partial compliance but also of changes in the drug metabolism, as it occurs in drug-drug interactions.

Other aspects suggested include non- or partial responders; in order to adjust dosage in those patients who have not reached full recovery after 3–4 weeks of treatment at therapeutic doses; or in patients that have many side effects with doses within the normal range (Cherma et al., 2009; Reis et al., 2004a; Reis et al., 2004b; Taurines et al., 2013). Compliance (the act of following a medical regimen or schedule correctly and consistently) has been evaluated through TDM in 2 studies in this review, showing that most patients did not follow their treatment schedule, which may cause the sertraline dosage to be subtherapeutic; and this reality worsens over the 6 months of follow-up. These authors also encourage taking an active role in verifying compliance, rather than a punitive one (Reis et al., 2004b). It is also emphasized that serum levels may help distinguish between poor adherence and rapid metabolism (Taurines et al., 2013). Other articles that have also evaluated this topic beyond this review show that it is mandatory actively look for the presence of side effects and to clearly identify the purpose of the treatment (specially in elderly people, (Cherma et al., 2009)). Furthermore, it is important to evaluate proper treatment adherence before changing treatment and that TDM may be a useful tool in order to follow these patients (who can also forget their daily requirements and therefore underestimate them (Reis et al., 2004a)). All of these potential utilities show that TDM, although not strictly necessary as a routine test in common clinical practice, has potential usefulness for strategies of personalized medicine.

Overall, the results of this scoping review are congruent with a non-linear relationship between the blood concentration of sertraline and its therapeutic and side effects. On the one hand, higher doses of sertraline are associated with higher serotonin transporter (SERT) occupancy in the brain (but non-linearly) (Hart et al., 2024), and greater antidepressant response (but not necessarily with poorer safety or tolerability, at least below 150 mg/day) (Luo et al., 2023). Of note, sertraline plasma concentration as low as 4.4 ng/mL has been associated with 80% SERT occupancy, whereas doses above 150 mg/day only lead to small increases in SERT occupancy in the order of 5% (Hart et al., 2024), which raises the question of whether further studies of sertraline TDM may refine the upper limit of the currently recommended range of 10–150 ng/mL (Hiemke et al., 2018) regarding efficacy. On the other one, while blood concentration increases proportionally with dose, interindividual variability may substantially modify this dose-concentration relationship due to CYP genotype, sex, age and hepatic function (Bråten et al., 2020; Castillo et al., 2024; De Vane et al., 2002; Jang and Jeong, 2024; Li et al., 2025). It may be worth noting that additional properties of sertraline such as its affinity for the sigma-1 receptor (Albayrak and Hashimoto, 2017) and possible DAT inhibition (Di Rocco et al., 1998) make it difficult to assess this question (Stahl, 2021). It may also be important to highlight that higher doses of SSRIs such as sertraline are commonly used and have been shown to be associated with improved response in OCD, a pattern that contrasts with major depressive disorder where increased doses do not necessarily improve outcomes (Bloch et al., 2010; Koran et al., 2007). This may also be explained by more complex mechanisms of sertraline beyond SERT inhibition.

Age may be a determinant of the diagnosis-specific utility of TDM, in view of the discrepancies regarding a possible association of sertraline serum concentration with clinical efficacy in pediatric OCD but not in pediatric MDD (Tini et al., 2022), and results indicating the possible usefulness of TDM in the search for the individual minimum effective dose in elderly patients treated with sertraline (Lundmark et al., 2000a).

Recommendations for future studies to assess the clinical utility of sertraline TDM include the incorporation of: i) randomized controlled trials; ii) comprehensive clinical assessments that capture the nuances of the psychopathological manifestations that underlie treatment response in each indication of sertraline, combining validated clinician-administered scales (such as, for instance, the HDRS, MADRS and HARS, together with suicide risk assessment using the Columbia-Suicide Severity Rating Scale (Posner et al., 2011) with patient reported outcomes of interest (such as the Beck Depression Inventory (Beck, 1961); iii) analysis of treatment response not based exclusively on total scores but on clinical domains derived from symptomatic profiles or selective items from clinical scales (for instance core depressive symptoms vs. physical, cognitive, anxiety, psychotic and suicidality related symptoms); iv) the interaction of TDM results with additional relevant factors associated with treatment response such as pharmacogenetic variables (including polymorphisms of CYP2C19 isoforms (Hicks et al., 2015) and possible photoconversion effects), drug interactions and relevant medical comorbidities (such as obesity and bariatric surgery); v) given the overlapping and difficult differentiation between some side effects and depressive symptoms, the use of the UKU-SERS in addition to patient self-reports may optimize the validity and sensitivity of the research of sertraline TDM to improve tolerability and safety (Panariello et al., 2023).

The strengths of this review include the wide range of ages, dosages, diagnoses and methodologies covered, and the diversity of the populations studied, encompassing a total of 1106 patients studied, that allow to map the evidence on the clinical utility of sertraline TDM. The findings of this review should be interpreted with caution given the gaps and limitations identified in this scoping review: quality and risk of bias of the included studies were not assessed, and the breadth and heterogeneity of the evidence covered preclude in-depth analyses and definitive conclusions. Furthermore, the studies included are mostly naturalistic with relatively uncontrolled conditions (Lundmark et al., 2000b; Mauri et al., 2002; 2003; Taurines et al., 2013; Tini et al., 2022), and many lacked of control group (Alderman et al., 1998; Lundmark et al., 2000b), randomization (Alderman et al., 1998; 2006), blinded evaluations (Alderman et al., 1998; Lundmark et al., 2000b) or control by possible cofounding factors (such as baseline severity, length of treatment and factors that can modify sertraline blood concentration, such as smoking habit and comedications), with three studies reporting efficacy data based only on global, non-specific clinical scales (Lundmark et al., 2000b; Taurines et al., 2013; Tini et al., 2022). Sampling time variability as a source of heterogeneity and lack of measures of the degree of compliance with the prescriptions of sertraline or missed doses reported by the participants as a limitation of the validity should be taken into account. Another limitation is that very different clinical entities such as MDD and OCD (which require different doses and lengths of treatments) were analyzed in single studies with a shared methodological approach. In addition, the sample and sub-sample sizes of the observational studies that yield concentration-effects are extremely low. Although this scoping review may serve a reference for further research on the clinical value of sertraline TDM, it should be emphasized that some methodological limitations of the studies included preclude robust evidence, such as lacking information regarding the specific type of biological matrix used in the TDM procedure, for instance that in the case of serum samples, sertraline concentration may decrease when stored in gel separator tubes compared with non-gel control tubes (Karppi et al., 2000; Shepard and Bliumkin, 2023). In order to maximize the reliability and control these limitations as much as possible, a preregistered protocol was drafted and the methodological recommendations from the JBI and PRISMA-ScR, reporting guidelines were followed.

## 5 Conclusion

To conclude, the current evidence on the concentration-effects relationships of sertraline does not justify a generalization of its TDM, and limits its clinical utility as guidance for clinical judgement in specific stages of treatment of MDD and OCD and populations with specific risks. All the blood level ranges estimations found in this review for efficacy (25–50 ng/mL for recurrent MDD in adults and 66–76 ng/mL for OCD in children and adolescents) and tolerability (44.8 ng/mL associated with side effects, *versus* 22.3 ng/mL) fall within the limits of the established consensus therapeutic range for the sertraline blood concentration, spanning from 10 to 150 ng/mL (Hiemke et al., 2018). There is preliminary evidence of the potential clinical utility of sertraline TDM to guide clinical decisions for personalized treatment optimization of MDD and OCD, especially in the adolescence and advanced age, in the presence of pharmacogenetic variants (especially CYP2C19), comorbidities or interactions that modify the metabolism of sertraline, and partial compliance. Future studies that incorporate the suggested recommendations may yield evidence to refine the current broad therapeutic range for specific ages, symptomatic profiles or clinical situations, and contribute further to the evidence on the potential clinical utility of sertraline TDM for personalized medicine.

## Data Availability

The original contributions presented in the study are included in the article/Supplementary Material, further inquiries can be directed to the corresponding authors.

## Author’s note

Pre-registered hypothesis and protocol at the Open Science Framework (https://osf.io/84trm/).
